# Frequency‐Dependent Seed Selection: How Relative Abundance and Seed Traits Jointly Mediate Foraging Preference in Scatter‐Hoarding Rodents

**DOI:** 10.1002/ece3.73611

**Published:** 2026-04-29

**Authors:** Kun Guo, Jinyu Zhang, Zhiyun Lu, Bo Wang

**Affiliations:** ^1^ School of Resources and Environmental Engineering Anhui University Hefei Anhui China; ^2^ School of Big Data and Statistics Anhui University Hefei Anhui China; ^3^ Ailaoshan Station of Subtropical Forest Ecosystem Studies, Xishuangbanna Tropical Botanical Garden Chinese Academy of Sciences Puer Yunnan China; ^4^ Anhui Province Key Laboratory of Wetland Ecosystem Protection and Restoration (Anhui University) Hefei Anhui China; ^5^ Anhui Shengjin Lake Wetland Ecology National Long‐Term Scientific Research Base Chizhou Anhui China

**Keywords:** frequency‐dependent selection, plant community dynamics, scatter‐hoarding rodents, seed dispersal, seed traits

## Abstract

Scatter‐hoarding rodents act as both seed predators and dispersers, strongly influencing seed fate and plant recruitment. Their foraging decisions are influenced not only by the traits of individual seeds but also by the traits of neighboring seeds. Although the effects of seed traits such as size and chemical defense are well‐established, how the relative frequency of co‐occurring seeds influences these decisions remains poorly understood. In this study, we conducted a field experiment in a subtropical forest in southwestern China using artificial seeds. A total of 19,200 artificial seeds with different seed sizes (diameters of 0.5, 1.5, and 2.5 cm) or tannin content (0%, 5%, and 10%) were deployed across 240 seed patches. Within each patch, the ratios of paired seeds were set across five levels (9:1, 7:3, 5:5, 3:7, and 1:9). We found that seed removal exhibited clear frequency‐dependent patterns. Specifically, large seeds were removed less frequently as their relative abundance increased, whereas small seeds showed the opposite trend when paired with larger seeds. Similar frequency‐dependent responses were observed for tannin treatments, although these effects were context‐dependent. In contrast, caching probability and dispersal distance remained largely unaffected by relative frequency. These results demonstrate that rodent foraging is jointly shaped by seed traits and frequency‐dependent processes, primarily during the initial removal stage. Our findings provide a mechanistic basis for understanding context‐dependent seed dispersal and highlight the role of frequency‐dependent selection in shaping plant community dynamics.

## Graphical abstract

This study demonstrates that scatter‐hoarding rodents exhibit frequency‐dependent foraging preferences for both seed size and tannin content. Large and high‐tannin seeds were less likely to be removed when abundant, while small and medium‐tannin seeds experienced increased removal at higher frequencies. These context‐dependent selection patterns highlight how relative seed abundance interacts with trait contrasts to shape seed predation and dispersal dynamics.

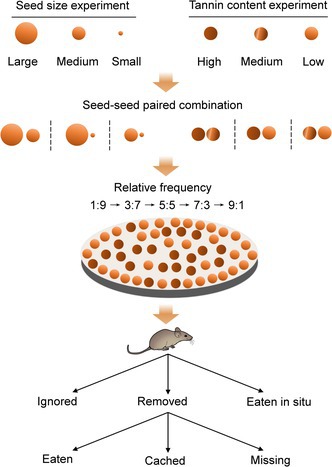

## 1 Introduction

As both seed predators and dispersers, scatter‐hoarding rodents play a pivotal role in mediating seed fate and seedling regeneration in many forest ecosystems (Vander Wall 2001; Steele et al. 2015; Lichti et al. 2017). Considerable variation in seed traits such as size and tannin content directly influences rodent foraging preferences, thereby determining whether seeds are consumed or dispersed (Vander Wall 2010; Jansen et al. 2012; Liu et al. 2023). During periods of fruit overlap, diverse tree species present rodents with seed assemblages that vary in these traits, to which rodents often exhibit distinct foraging and caching preferences (Moore et al. 2007; Wang et al. 2012; Yang et al. 2020). Importantly, these preferences are affected not only by intrinsic seed traits but also indirectly by the traits of neighboring seeds, which is known as the associational or neighborhood effect (Ostoja et al. 2013; Lichti et al. 2014; Xiao et al. 2022; He et al. 2025).

A growing body of evidence demonstrates that co‐occurring seeds can significantly influence foraging decisions for a target seed, leading to apparent competitive or mutualistic interactions. For example, Xiao et al. (2022) found that neighboring seeds with contrasting traits (tannin content, seed size, and dormancy) significantly influenced seed removal, dispersal, and dispersal distance through trait‐mediated indirect interactions. Similarly, Liu et al. (2023) reported that contrasting seed size between neighboring seeds significantly affected rodent foraging behavior by increasing removal and caching of larger seeds, whereas contrasts in tannin and nutrient content had limited influence. However, these insights largely derive from experiments using fixed and often equal ratios of paired seeds. In nature, the heterogeneous distribution of plant communities creates a mosaic of foraging patches characterized by dynamic and unequal proportions of different seed traits (Shimada et al. 2015; Zhang and Wang 2025). Although the effects of neighboring seeds at equal ratios are increasingly understood, the ecological consequences of their dynamic relative frequencies remain largely unexplored.

Frequency‐dependent selection represents a fundamental mechanism in ecology, whereby the fitness of a phenotype depends on its relative abundance within a population or community (Allen et al. 1998). Negative frequency‐dependent selection, in particular, can promote the maintenance of trait diversity by conferring advantages to rare phenotypes (Horst and Venable 2018). In plant–animal interactions, such processes may emerge when consumers disproportionately target common resource types, thereby reducing their relative success and allowing less common types to persist. Frequency‐dependent foraging has the potential to generate dynamic feedbacks between seed traits and plant recruitment (Zhang et al. 2024). By selectively removing or caching seeds based on their relative abundance, rodents may influence not only individual seed fate but also the distribution of functional traits within plant communities (Zhang et al. 2023). Despite its importance, empirical tests of frequency‐dependent selection in seed dispersal systems remain limited, particularly in relation to trait‐mediated neighborhood effects.

Research on frequency‐dependent effects in scatter‐hoarding rodents has advanced through diverse experimental approaches. At the community level, evidence from tropical systems indicates that the aggregate seed density of co‐occurring species can be a stronger predictor of predation than conspecific density, underscoring the role of shared rodent predators in mediating complex plant‐community dynamics (Garzon‐Lopez et al. 2015). Several studies have manipulated seed trait ratios to examine the influence of relative frequency on foraging preferences, yet findings show inconsistencies. Early work by Hulme and Hunt (1999) found that strong inherent preferences in rodents overrode frequency effects across the tested ratios, although their scope was limited to seed removal. In contrast, Zhang et al. (2024) reported that tannin content determines the choice between caching and consumption, whereas the relative frequency of seed sizes exerts a positive frequency‐dependent influence on rodent foraging decisions. These studies have typically relied on simplified experimental designs that overlook the inherent complexity of seeds. Seeds possess multiple co‐occurring traits, such as size, nutrient content, and chemical and physical defenses, that collectively influence rodent foraging decisions (Lichti et al. 2017; Wang et al. 2018). Moreover, such designs often overlook the substantial intraspecific variation within these traits (Shimada et al. 2015; Wang and Ives 2017; Ding et al. 2026). Consequently, the expression of associational effects is likely highly context‐dependent, varying with the specific trait values of individual seeds and complicating accurate predictions of foraging outcomes in natural systems.

Artificial seeds provide a key experimental approach for isolating the effect of a single trait, enabling researchers to manipulate that trait while holding all others constant, and thus assess its specific influence on behavioral responses (Wang and Chen 2009). For example, using this method, Wang (2020) demonstrated that neighborhood effects between high‐ and low‐tannin seeds significantly shape rodent foraging trade‐offs between immediate consumption and scatter‐hoarding. Recent studies employing artificial seeds have further clarified the complex interactions between seed traits and neighborhood context, revealing that trait contrasts between a target seed and its neighbors can significantly alter seed fate, resulting in outcomes that encompass a continuum from apparent mutualism to apparent competition (Liu et al. 2023). However, a critical limitation is that these insights largely stem from experiments using simplified and fixed (e.g., 1:1) seed ratios. Such designs fail to capture the variable and often highly skewed distributions of co‐occurring seeds with different traits found in natural seed patches, thereby limiting their ability to test the full scope of trait‐mediated neighborhood effects.

To investigate frequency‐dependent foraging preferences in scatter‐hoarding rodents, we conducted a field experiment using artificial seeds. We focused on two traits central to rodent foraging: seed size and tannin content. For each paired trait combination, we tested them across a gradient of five relative frequencies (9:1, 7:3, 5:5, 3:7, and 1:9). Specifically, we addressed two questions: (1) How does the relative frequency of co‐occurring seeds influence rodent selection at different stages of the dispersal process? (2) Is the effect of relative frequency consistent across different types of trait contrasts (seed size and tannin content)? By integrating relative frequency with trait‐mediated neighborhood effects and examining them across the foraging stages, this study advances a mechanistic understanding of how context‐dependent selection operates in rodent–seed interactions, with implications for seed dispersal dynamics and plant community assembly.

## 2 Material and Methods

### 2.1 Study Site

This study was conducted within the Ailao Mountains National Nature Reserve in Yunnan Province, southwestern China (24°32′ N, 101°01′ E, elevation 2045 m). The site comprises a mid‐elevation subtropical evergreen broad‐leaved forest. The regional climate is characterized by a mean annual temperature of 11.3°C and a mean annual precipitation of 1931 mm. The forest is dominated by tree species of *Lithocarpus hancei*, *Lithocarpus xylocarpus*, and *Castanopsis wattii*, which primarily rely on small rodents for seed dispersal (Wen et al. 2018). Key disperser species in this system include *Niviventer confucianus* , *Apodemus draco* , and *Niviventer excelsior* (Xiao and Zhang 2012; Liu et al. 2023; Wang et al. 2025).

### 2.2 Artificial Seeds

Artificial seeds were prepared from a mixture of peanut powder, clay, and tannin (Zhiyuan Chemical Reagent Co., Tianjin, China). Following the method of Wang and Chen (2009), each seed was molded into a sphere and attached with a thin metal wire to a plastic identification tag (3.5 cm × 2.5 cm) to permit individual tracking. We manipulated seed traits in two independent experimental series: size and tannin content. Size levels were small (0.5 cm diameter), medium (1.5 cm), and large (2.5 cm); tannin levels were low (0%), medium (5%), and high (10%). In the size series, seeds were prepared with a 1:1 peanut‐powder‐to‐clay ratio and contained no tannin. In the tannin series, seed diameter was fixed at 1.5 cm, and the base mixture consisted of equal parts peanut powder and clay, with tannin added to achieve the target concentrations. These trait levels were selected to reflect the natural interspecific variation observed in local plant species, based on prior studies (Wang and Chen 2009; Wang et al. 2012; Wang 2020).

### 2.3 Field Experiment

**FIGURE 1:**
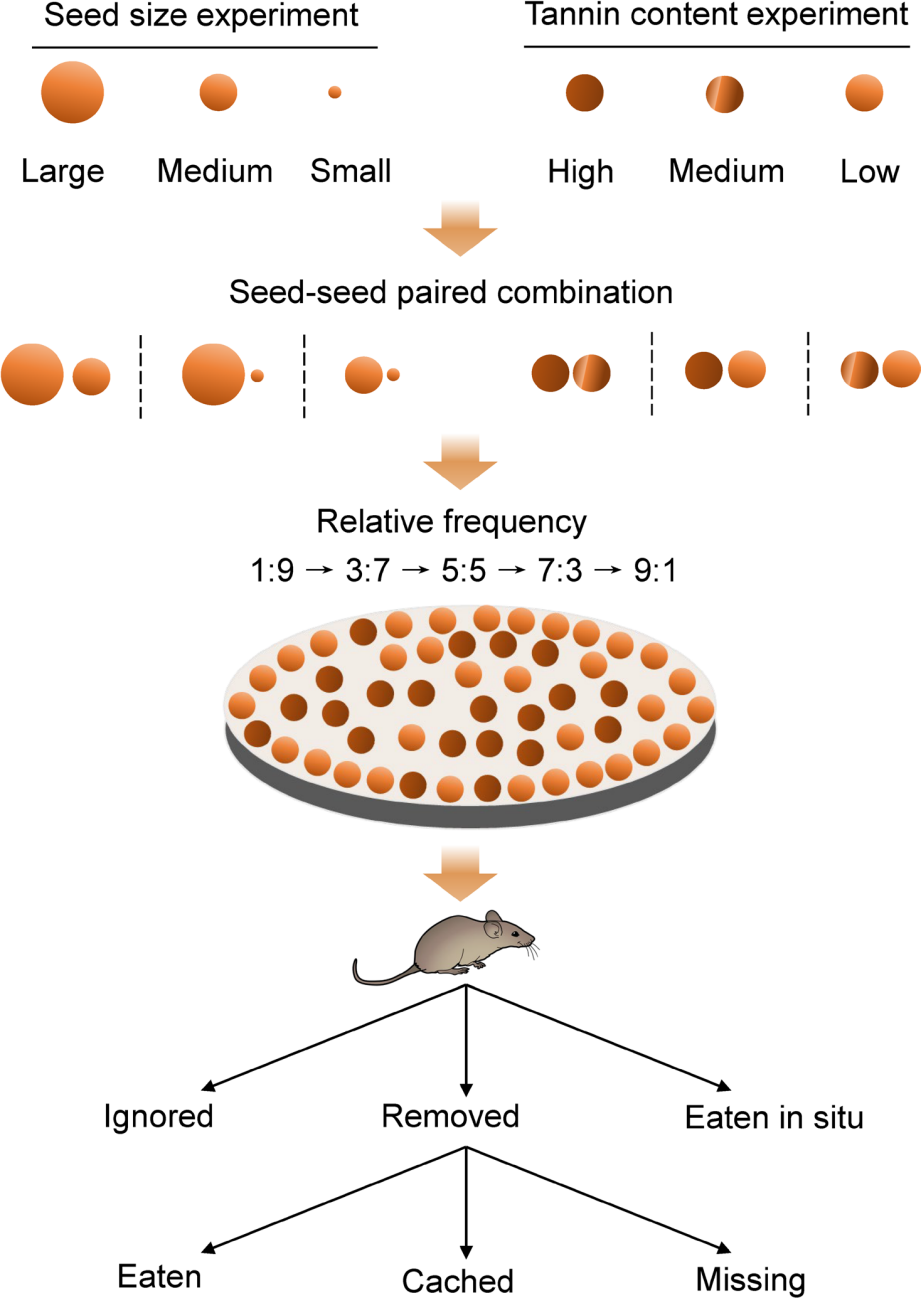
Experimental design illustrating paired‐seed treatments and relative frequency gradients used to assess rodent foraging behavior. Artificial seeds differing in either size (large, medium, small) or tannin content (high, medium, low) were arranged in pairwise combinations. Within each pair, seeds were presented across five relative frequency treatments (1:9, 3:7, 5:5, 7:3, 9:1). Seed fates were recorded as ignored, removed, eaten in situ, eaten after removal, cached, or missing. This design allowed us to test how relative abundance and seed traits jointly influence different stages of the seed dispersal process.

In late November 2024 and early April 2025, we established a total of 240 seed release points (120 per year), spaced at least 30 m apart, within the study forest. The timing of the experiment was chosen to avoid interference from the natural fruiting season (August to November) and to minimize background seed availability. Using a paired‐seed design to control relative frequency within patches, we tested all pairwise combinations of the three trait levels for seed size (large vs. small, large vs. medium, medium vs. small) and tannin content (high vs. low, high vs. medium, medium vs. low). At each release point, 80 artificial seeds were placed in a circular arrangement according to the specified ratio for paired treatments. Each paired combination was tested at five frequency ratios: 1:9, 3:7, 5:5, 7:3, and 9:1 (Figure 1). For each trait, the experiment comprised a total of 15 treatments, which included all frequency variations across the three paired combinations. The attached plastic tags were oriented radially outward to facilitate relocation. The entire design was replicated four times annually at different, randomly assigned release points. Over 2 years and across both trait series (seed size and tannin content), a total of 19,200 artificial seeds were deployed (80 seeds × 15 treatments × 4 replicates × 2 traits × 2 years).

### 2.4 Seed Fate Survey

The fates of artificial seeds were assessed at 1, 2, 3, 4, 6, 8, 12, and 16 days after release. During each survey, systematic searches were conducted within a 30‐m radius of each release point. This distance exceeds the typical seed‐dispersal range (< 20 m) of the local rodent community within the selected forest (Feng et al. 2021; Chen et al. 2022; Liu et al. 2023). Extended searches beyond this radius were also performed up to a maximum distance of 50 m to locate seeds dispersed farther away. Seed fate was initially categorized as ignored (left intact at the release point), eaten in situ, or removed. Removed seeds were subsequently classified as eaten, cached (intact and hoarded in the soil or on the forest floor), or missing (not recovered, with final fate undetermined). For removed seeds, the straight‐line dispersal distance from the transport location to the original release point was recorded, but those that were not relocated were excluded from analyses because their final positions were unknown.

### 2.5 Data Analysis

This study analyzed three key seed‐fate variables: (1) removal probability (the proportion of seeds removed from the release point), (2) caching probability (the proportion of removed seeds that were cached), and (3) dispersal distance of removed seeds. To evaluate the influence of relative seed frequency on rodent foraging decisions, we used generalized linear mixed models (GLMMs) with a binomial error distribution to analyze the probabilities of seed removal and caching, and GLMMs with a Gamma error distribution were used to analyze dispersal distances. Within the seed size and tannin content experiments, we analyzed each target seed separately, fitting independent models for each paired comparison. In all models, the relative frequency of the target seed within an experimental patch was fitted as a fixed effect, while release point identity and experimental year were included as random intercepts. To account for potential non‐linear biological patterns, we additionally fitted models including both linear and quadratic terms of relative frequency, and the model with the minimum Akaike's Information Criterion (AIC) was considered as the final model. Residual diagnostics and dispersion tests were conducted to assess model adequacy using the “DHARMa” package, with all models meeting assumptions. To avoid the risk of Type I error, we applied a false discovery rate correction using the Benjamini–Hochberg procedure to adjust *p*‐values. For all models, we also calculated marginal and conditional *R* ^2^ values using the “MuMIn” package. All statistical analyses were conducted using R software version 4.4.2 (R Core Team 2025).

## 3 Results

### 3.1 Overall Patterns of Seed Fate

In this experiment, 98.84% (18,977) of all artificial seeds were harvested; only 1.16% (223) of seeds remained ignored. In the seed size experiment (*n* = 9600), 21.93% (2105) of seeds were removed, 77.00% (7392) were eaten in situ, and 1.07% (103) were ignored. Of the removed seeds, 3.26% (313) were cached, while 1.69% (1162) were missing. The mean dispersal distance of removed seeds was 2.79 ± 0.05 m (mean ± SE), with a maximum distance of 17.22 m. In the tannin experiment (*n* = 9600), 15.01% (1441) were removed by rodents, 83.74% (8039) were eaten in situ, and the remaining 1.25% (120) were left intact at their original release points. Among removed seeds, 2.68% (257) were cached, and 2.79% (268) were missing. The mean dispersal distance of removed seeds was 2.46 ± 0.047 m, with a maximum distance of 11.79 m.

### 3.2 Frequency‐Dependent Effects on Seed Fate

**FIGURE 2:**
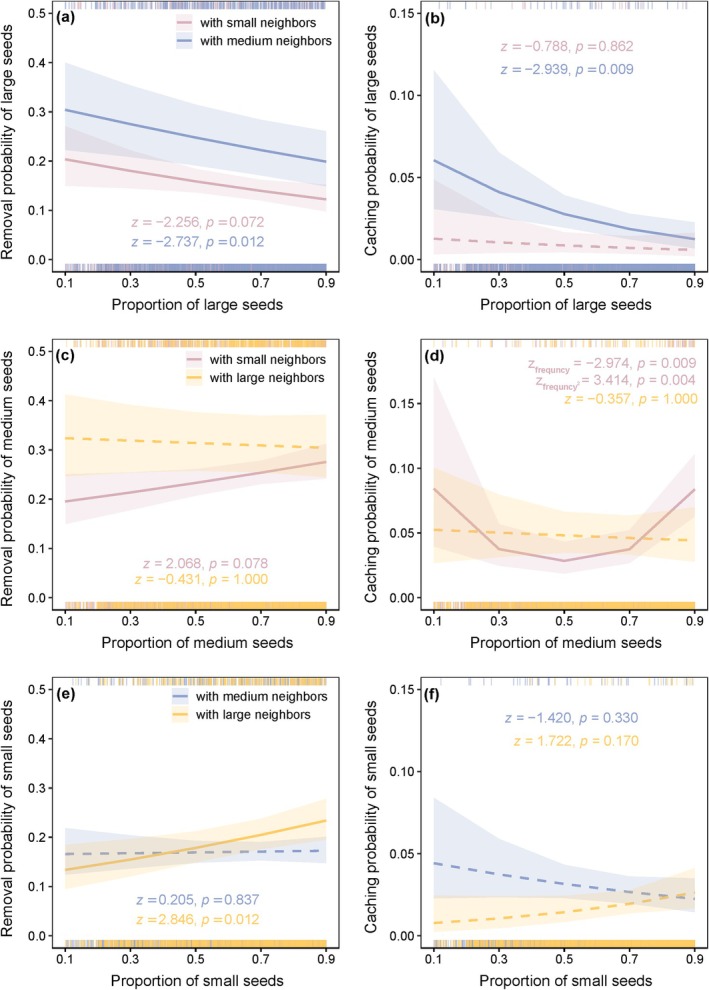
Effects of relative frequency on seed removal and caching probabilities across seed‐size pairings. The relationships between the relative frequency of target seeds and their probability of removal (left panels) and caching (right panels) are shown for: (a, b) large seeds paired with medium or small seeds; (c, d) medium seeds paired with large or small seeds; (e, f) small seeds paired with large or medium seeds. Shaded bands represent 95% confidence intervals. Detailed statistical results are presented in Table 1.

Large seeds showed a significant decrease in removal probability as their proportion increased when paired with medium seeds (*z* = −2.737, *p* = 0.012) and small seeds (*z* = −2.256, *p* = 0.072; Figure 2a). Similarly, the caching probability of large seeds decreased with higher proportions when paired with medium seeds (*z* = −2.939, *p* = 0.009), but not when paired with small seeds (*z* = −0.788, *p* = 0.862; Figure 2b). For medium seeds, removal probability increased with their proportion when paired with small seeds (*z* = 2.068, *p* = 0.078), but not when paired with large seeds (*z* = −0.431, *p* = 1.000; Figure 2c). Notably, the caching probability of medium seeds showed a U‐shaped relationship with proportion when paired with small seeds, but no significant variation when paired with large seeds (Figure 2d). The removal probability of small seeds increased with proportion when paired with large seeds (*z* = 2.846, *p* = 0.012), but not when paired with medium seeds (*z* = 0.205, *p* = 0.837; Figure 2e); caching probability of small seeds was not influenced in any pairings (Figure 2f). Moreover, the relative frequency of seed size showed no significant effect on dispersal distance across all treatments (all *p* > 0.05; Table 1).

**TABLE 1:** Results of generalized linear mixed model (GLMMs) examining the effects of target seed relative frequency on the probability of removal, caching, and dispersal distance for each pairing in the seed‐size experiment.

Seed fate	Estimate	SE	*z*/*t*‐value	*p*. adjust	*R* ^2^m	*R* ^2^c
Large seeds paired with medium seeds						
Removal (1600)	−0.707	0.258	−2.737	0.012	0.01	0.02
Caching (1600)	−2.036	0.693	−2.939	0.009	0.06	0.11
Dispersal distance (341)	−0.111	0.215	−0.515	0.607	0.00	0.32
Large seeds paired with small seeds						
Removal (1600)	−0.759	0.336	−2.256	0.072	0.01	0.03
Caching (1600)	−0.976	1.239	−0.788	0.862	0.01	0.28
Dispersal distance (191)	0.101	0.315	0.321	0.862	0.00	0.12
Medium seeds paired with large seeds						
Removal (1600)	−0.114	0.263	−0.431	1.000	0.00	0.02
Caching (1600)	−0.225	0.631	−0.357	1.000	0.00	0.11
Dispersal distance (470)	0.255	0.175	1.452	0.441	0.01	0.03
Medium seeds paired with small seeds						
Removal (1600)	0.562	0.272	2.068	0.078	0.01	0.01
Caching (1600)					0.06	0.07
Frequency	−7.144	2.402	−2.974	0.009		
Frequency^2^	7.135	2.089	3.414	0.004		
Dispersal distance (377)	0.302	0.207	1.462	0.144	0.01	0.08
Small seeds paired with large seeds						
Removal (1600)	0.852	0.299	2.846	0.012	0.01	0.02
Caching (1600)	1.544	0.897	1.722	0.170	0.04	0.04
Dispersal distance (306)	−0.006	0.367	−0.017	0.986	0.00	0.14
Small seeds paired with medium seeds						
Removal (1600)	0.059	0.286	0.205	0.837	0.00	0.00
Caching (1600)	−0.873	0.615	−1.420	0.330	0.01	0.01
Dispersal distance (258)	−0.433	0.271	−1.597	0.330	0.01	0.08

*Note:* The numbers indicate the sample size analyzed for each model. *R* ^2^m, marginal *R* ^2^; *R* ^2^c, conditional *R* ^2^; *p*. adjust, adjusted *p*‐values.

**FIGURE 3:**
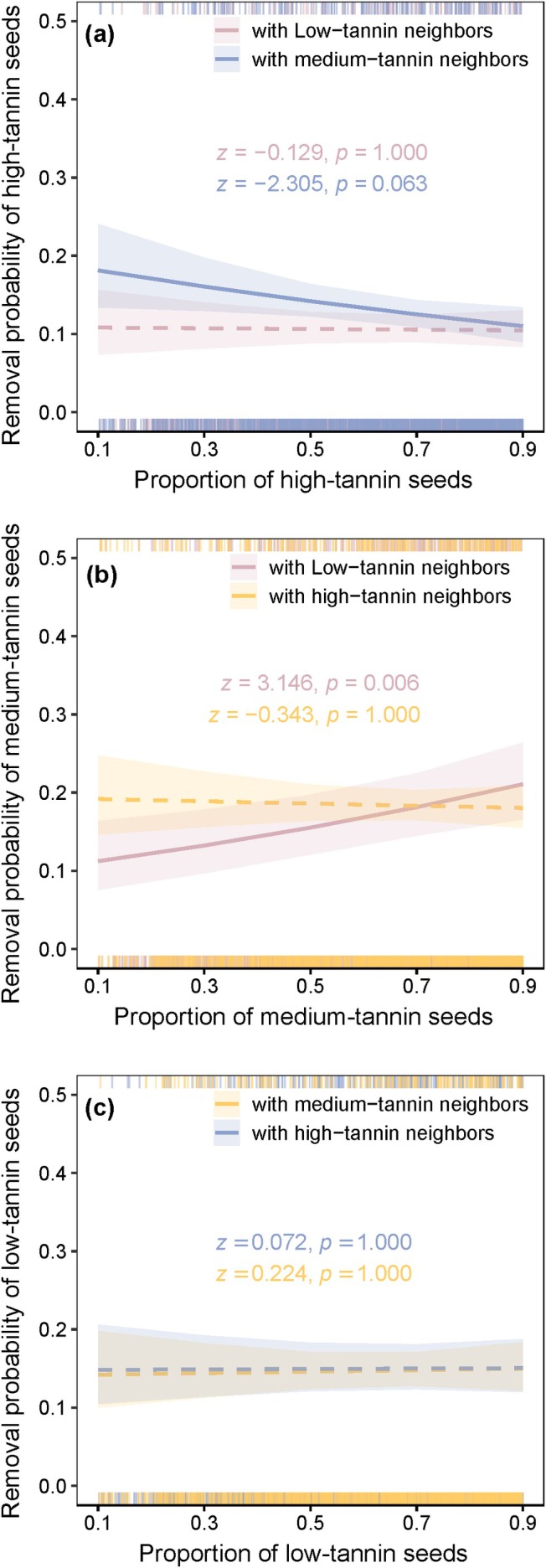
Effects of relative frequency on seed removal probability across tannin‐content pairings. The relationship between the relative frequency of target seeds and their removal probability is shown for: (a) high‐tannin seeds paired with medium‐ or low‐tannin seeds; (b) medium‐tannin seeds paired with high‐ or low‐tannin seeds; (c) low‐tannin seeds paired with high‐ or medium‐tannin seeds. Shaded bands represent 95% confidence intervals. Detailed statistical results are presented in Table 2.

In the tannin experiment, the removal probability of high‐tannin seeds decreased with increasing proportion when paired with medium‐tannin seeds (*z* = −2.305, *p* = 0.063), but not when paired with low‐tannin seeds (*z* = −0.129, *p* = 1.000; Figure 3a). The removal probability of medium‐tannin seeds increased with their proportion when paired with low‐tannin seeds (*z* = 3.146, *p* = 0.006), but not when paired with high‐tannin seeds (*z* = −0.343, *p* = 1.000; Figure 3b). The proportion of low‐tannin seeds showed no significant effect on their removal probability in any pairing (Figure 3c). Caching probability was not significantly influenced by relative frequency for any tannin‐level comparison, and dispersal distance was also unaffected (all *p* > 0.05; Table 2). Overall, seed combinations containing medium‐sized and medium‐tannin seeds exhibited higher removal rates and longer dispersal distances than other combinations.

**TABLE 2:** Results of generalized linear mixed model (GLMMs) examining the effects of target seed relative frequency on the probability of removal, caching, and dispersal distance for each pairing in the tannin‐content experiment.

Seed fate	Estimate	SE	*z*/*t*‐value	*p*. adjust	*R* ^2^m	*R* ^2^c
High‐content seeds paired with medium‐content seeds						
Removal (1600)	−0.729	0.316	−2.305	0.063	0.01	0.01
Caching (1600)	−0.834	0.674	−1.237	0.216	0.01	0.11
Dispersal distance (155)	−0.472	0.241	−1.960	0.100	0.04	0.09
High‐content seeds paired with low‐content seeds						
Removal (1600)	−0.046	0.354	−0.129	1.000	0.00	0.00
Caching (1600)	0.176	0.852	0.206	1.000	0.00	0.08
Dispersal distance (124)	0.024	0.399	0.061	1.000	0.00	0.14
Medium‐content seeds paired with high‐content seeds						
Removal (1600)	−0.095	0.276	−0.343	1.000	0.00	0.00
Caching (1600)	0.513	0.590	0.870	1.000	0.00	0.08
Dispersal distance (259)	0.060	0.235	0.253	1.000	0.00	0.09
Medium‐content seeds paired with low‐content seeds						
Removal (1600)	0.933	0.297	3.146	0.006	0.01	0.02
Caching (1600)	0.081	0.822	0.098	0.922	0.00	0.00
Dispersal distance (234)	0.324	0.290	1.119	0.526	0.01	0.14
Low‐content seeds paired with high‐content seeds						
Removal (1600)	0.078	0.347	0.224	1.000	0.00	0.02
Caching (1600)	0.211	0.637	0.331	1.000	0.00	0.11
Dispersal distance (199)	0.267	0.258	1.010	0.936	0.01	0.11
Low‐content seeds paired with medium‐content seeds						
Removal (1600)	0.022	0.300	0.072	1.000	0.00	0.00
Caching (1600)					0.04	0.11
Frequency	8.397	4.925	1.738	0.252		
Frequency^2^	−7.349	3.946	−1.863	0.252		
Dispersal distance (202)	−0.155	0.287	−0.541	1.000	0.00	0.09

*Note:* The numbers indicate the sample size analyzed for each model. *R* ^2^m, marginal *R* ^2^; *R* ^2^c, conditional *R* ^2^; *p*. adjust, adjusted *p*‐values; #, marginal significant.

## 4 Discussion

This study demonstrates that the relative frequency of co‐occurring seeds significantly influences rodent foraging preferences, revealing distinct patterns of frequency‐dependent selection for both seed size and tannin content. These results indicate that rodent foraging decisions are not only trait‐based but also strongly context‐dependent, with the direction and strength of frequency‐dependent effects depending on both trait contrast and the specific stage of the dispersal process. Our findings support and extend previous research on associational effects in rodent‐seed interactions, highlighting that the relative abundance of seeds with distinct traits can create dynamic selection mosaics that shape plant seed fate, transcending simple trait‐mediated preferences.

Seed size showed strong and consistent frequency‐dependent effects on rodent foraging decisions. In our experiment, the removal probability of large seeds decreased as their relative frequency increased when paired with medium or small seeds, whereas small seeds exhibited the opposite pattern when paired with large seeds. These results indicate that rodents adjust their foraging behavior in response to the relative abundance of energetically contrasting resources. Such patterns are consistent with previous studies showing that larger seeds are generally preferred due to higher energetic returns (Jansen et al. 2004; Xiao et al. 2015), but this preference can weaken when large seeds become abundant. One possible explanation is that handling‐time constraints may limit the efficient exploitation of abundant large seeds, leading rodents to shift toward smaller, more easily processed seeds. This interpretation aligns with optimal foraging theory, which predicts that consumers balance energy gain with foraging costs (Kotler et al. 1999; van der Merwe et al. 2014).

In contrast, frequency‐dependent effects associated with tannin content were more context‐dependent and less consistent across pairings. In our study, high‐tannin seeds showed reduced removal at higher relative frequencies when paired with medium‐tannin seeds, whereas medium‐tannin seeds were removed more frequently as their proportion increased when paired with low‐tannin seeds. These results suggest that the influence of chemical defenses on foraging decisions depends strongly on the specific trait contrast within the seed assemblage. Compared with seed size, tannin content may be less immediately detectable by rodents and may require sampling or learning before influencing foraging decisions. This could delay or weaken frequency‐dependent responses. Previous studies have similarly reported that chemical traits often exert weaker or more variable effects on rodent foraging than physical traits such as seed size (Wang and Chen 2009; Kuprewicz and García‐Robledo 2019). Our results extend these findings by showing that the strength of frequency dependence itself can vary depending on the type of trait involved.

Our results indicate that caching probability showed weak or no frequency dependence across treatments in contrast to removal. This pattern may arise from multiple, non‐mutually exclusive mechanisms. First, caching decisions may depend more strongly on intrinsic seed traits, such as nutritional value and perishability, than on their relative abundance within a patch (Gómez et al. 2008). Second, rodents may experience satiation effects during the removal stage, reducing the influence of frequency on subsequent hoarding decisions. Finally, trade‐offs between predation risk and cache protection may constrain flexible responses to frequency, as caching often occurs under different ecological conditions than initial seed selection. Together, these factors suggest that frequency‐dependent processes may operate more strongly during initial foraging decisions than during post‐removal handling stages.

The different responses to frequency in the seed size and tannin experiments indicate that associational effects are trait‐specific. Seed size, being easily detectable by rodents and directly linked to energy gain, resulted in strong and consistent frequency‐dependent removal across all seed pairings. In contrast, the influence of tannin content was more contingent on the specific pairing, with clear frequency‐dependent responses observed only under certain conditions, such as high‐ versus medium‐tannin seeds. This divergence may stem from the fact that chemical defenses like tannins are not visually apparent; rodents likely need to sample or learn to recognize them, a process that could delay frequency‐based foraging decisions (Bozinovic and Novoa 1997; Steele et al. 2001). Moreover, frequency showed no significant effect on caching behavior in the tannin experiment. This implies that once a seed is removed, the decision to cache or consume it depends more on its palatability or nutritional value than on its initial abundance. These findings are consistent with previous studies demonstrating that seed size often exerts a stronger influence on rodent foraging than chemical traits (Wang and Chen 2009; Kuprewicz and García‐Robledo 2019). Nevertheless, they also underscore how trait interactions and frequency dynamics can mediate such hierarchical relationships.

Frequency‐dependent foraging may promote seed‐trait diversity and species coexistence in forest ecosystems. By disproportionately harvesting common seeds while sparing rarer ones, scatter‐hoarding rodents can reduce the dominance of any single trait strategy, thereby maintaining a diversity of seed traits within and among plant populations. Furthermore, the stage‐specific nature of frequency effects implies that seed survival and dispersal are shaped by a sequence of context‐dependent decisions. This hierarchical decision‐making process could create differential recruitment opportunities for different plant species under varying competitive scenarios, contributing to the maintenance of plant community diversity (Sundaram et al. 2017; Zhang et al. 2023; DeFilippis et al. 2025).

Although our use of artificial seeds allowed us to isolate the effect of relative frequency for individual traits, this approach simplifies the natural complexity of real seeds. In nature, seeds typically present a combination of linked traits, such as size, tannin levels, nutrient content, and physical defenses. These traits work together to shape rodent foraging decisions (Lichti et al. 2017; Wang et al. 2018). We observed clear frequency‐dependent selection in our single‐trait experiments, which suggests the same mechanism likely functions in more complex, multi‐trait settings. Future studies should manipulate combinations of traits across frequency gradients to better clarify how frequency‐dependent selection works within more realistic trait combinations. Furthermore, our research focused on seed removal and caching because they are critical determinants of seedling establishment. The frequency‐dependent patterns observed at the seed stage are likely to carry over to later life stages, potentially influencing the spatial distribution of seedling emergence and altering the composition of regenerating plant communities. A related limitation is that seeds removed but not relocated were excluded from dispersal distance analyses; therefore, our estimates are based only on recovered seeds and may underestimate true dispersal distances, particularly for rare long‐distance dispersal events. Long‐term experiments that link frequency‐dependent seed removal with seedling survival and growth would be highly valuable. Such studies are essential to elucidate how these early‐stage filtering mechanisms scale up to influence plant population and community dynamics.

In conclusion, this study provides experimental evidence that rodent foraging decisions are shaped by both trait‐based preferences and frequency‐dependent selection. Our findings highlight the importance of frequency‐dependent foraging as a potential mechanism linking individual‐level consumer behavior to community‐level trait dynamics. By disproportionately targeting common resource types, scatter‐hoarding rodents may generate negative frequency‐dependent selection on seed traits, thereby contributing to the maintenance of trait diversity in plant communities. This mechanism provides a behavioral pathway through which consumer decisions can scale up to influence plant coexistence and community assembly. Importantly, our results suggest that such frequency‐dependent effects are most pronounced during the initial removal stage, whereas later stages may be governed by additional ecological constraints. Future work ought to be expanded by incorporating multiple interacting traits, natural seed assemblages, and longer‐term fitness consequences to fully unravel the ecological and evolutionary implications of frequency‐dependent dispersal.

## Author Contributions

**Kun Guo:** data curation (lead), formal analysis (equal), investigation (lead), software (equal), visualization (equal), writing – original draft (equal). **Jinyu Zhang:** formal analysis (equal), funding acquisition (equal), methodology (equal), resources (equal), software (equal), supervision (equal), validation (equal), visualization (equal), writing – original draft (lead), writing – review and editing (lead). **Zhiyun Lu:** formal analysis (equal), investigation (equal), writing – original draft (equal). **Bo Wang:** conceptualization (equal), funding acquisition (equal), project administration (equal), resources (equal), supervision (equal), writing – original draft (equal), writing – review and editing (equal).

## Funding

This study was funded by the National Natural Science Foundation of China (32171533 and 31971444), the China Postdoctoral Science Foundation (2025M772836), and the Postdoctoral Fellowship Program of CPSF (GZC20251683).

## Conflicts of Interest

The authors declare no conflicts of interest.

## Data Availability

All the data used in the manuscript have been submitted to the Dryad Digital Repository, and have been assigned a unique digital object identifier (DOI): https://doi.org/10.5061/dryad.g79cnp64s.
